# Factors associated with work volition among Chinese undergraduates

**DOI:** 10.3389/fpsyg.2022.1037185

**Published:** 2022-12-21

**Authors:** Lu Hai, Xiaohong Bao, Wenyu Li

**Affiliations:** School of Education, Minzu University of China, Beijing, China

**Keywords:** work volition, economic constraints, ethnic identity, minzu, hukou, Zhongyong thinking

## Abstract

Based on the psychology of working theory, this study tested the relationships among work volition and the predictors of economic constraints, minzu (ethnic identity), Hukou and Zhongyong thinking, and determined how Hukou and Zhongyong thinking moderate the relationship between economic constraints and work volition with a sample of 2,995 undergraduates in China. The results showed that work volition was negatively associated with economic constraints but positively related to Zhongyong thinking. The results further revealed that Hukou and Zhongyong thinking were significant moderators in the link between economic constraints and work volition, while weaker negative associations were found between economic constraints and work volition for undergraduates who came from rural areas and had lower levels of Zhongyong thinking. Implications for research and practice are discussed.

## 1. Introduction

Work volition refers to the perceived capacity to make occupational choices (or future occupational decisions for college students) despite constraints (Duffy et al., 2012), which might be viewed as having important positive implications, such as career adaptability, decent work, career decision-making self-efficacy, academic satisfaction, job satisfaction, and work locus of control (Jadidian and Duffy, 2012; Duffy et al., 2013, 2015, 2016a). Therefore, this topic has gained more attention over the last several years (Duffy et al., 2015). In addition, work volition is malleable (Duffy et al., 2016a); therefore, it is necessary to analyze its related predictors and control it effectively. Several studies documented how recent health symptoms, illness perceptions, social support, subjective social status, acculturative stress, workplace climate, economic constraints, and marginalization predict work volition (Kim et al., 2018, 2022; Wang et al., 2019; England et al., 2020; Bouchard and Nauta, 2021).

However, previous research has several limitations, one of which is a lack of cultural perspectives. As Berry et al. (2011) suggested, there are common psychological processes in all humans, but culture shapes the development and expression of these underlying features. Thus, the mechanism of work volition should be considered from both common and unique cultural paths; however, few studies have taken both into account, with the latter being particularly neglected. Previous studies found that, compared to college students from other countries, Chinese undergraduate students on average report lower work volition levels while facing economic constraints (Cheung et al., 2020), which may be understood in depth and accurately from Chinese cultural factors. Therefore, to balance and draw a clearer picture of working psychology in the Chinese context, this study is the first to use certain scales to measure the level of work volition in Chinese college students, considering the relationships among economic constraints, minzu (ethnic identity), hukou (registered permanent residence), and Zhongyong thinking, and work volition. Economic constraints are a group of variables that are supported by studies on work volition in both Eastern and Western cultures (e.g., Cheung et al., 2020); however, the other three variables have not received research attention in this field before and are proposed in this study based on the Chinese context and culture. Although previous studies included ethnic identity, the concept has a unique meaning in China. Therefore, in this study, minzu (ethnic identity) is regarded as a new variable within the Chinese cultural context. Hardin et al. (2014) indicated that psychological science will benefit by adopting approaches that more accurately represent our understanding of the complex interplay of universal and culturally specific influences on psychological phenomena. Thus, we believed that this study will not only be helpful in further confirming the relationship between commonality factors and work volition but also attempted to expand the current knowledge on the psychology of working theory (PWT) in specific countries and cultures, thereby eventually leading to an inclusive and integrative psychology of working (Blustein, 2001).

## 2. Theoretical framework

Many theories in vocational psychology (e.g., typology theory and social cognitive career theory) have been proposed to explore essential psychological elements of career development and work; however, some problems exist. For example, they paid more attention to the work experience of the middle class and did not adequately explain the work-based experiences of people on the ‘lower rungs of the social position ladder'—people without sufficient access to financial and social capital, marginalized people (i.e., who are marginalized based on factors such as race, ethnicity, social class, and/or gender), and people who are forced to make involuntary work-based transitions (Duffy et al., 2016a). This can result in the misunderstanding that individuals can choose the careers they will pursue (Jadidian and Duffy, 2012). In addition, these theories have primarily focused on personal traits such as values, skills, and interests in career decision-making and secondarily on the context of the life of an individual (Jadidian and Duffy, 2012). Faced with these dilemmas, the Psychology of Working Framework (PWF) and PWT were developed in succession, with the primary focus of both being to document the work lives of those with limited access to opportunity and to indicate there are a variety of constraints that can limit the perception of volition in their decision-making for most individuals. These constraints include, but are not limited to, poverty, financial stress, physical disabilities, mental disabilities, family pressures, and discrimination (Duffy et al., 2014). Moreover, they highlight contextual factors that are more relevant to underrepresented populations (Duffy et al., 2016a, 2019). However, PWF does not contain specific, empirically testable propositions or outline how a wide array of factors can collectively promote work experiences. Thus, these gaps were filled by PWT, which offers a new, empirically testable model (Duffy et al., 2019). In this model, it identified two basic contextual factors (economic constraints and marginalization), two mediator variables (work volition and career adaptability), and four moderator variables (proactive personality, critical consciousness, social support, and economic conditions) that may alter the direction and strength of contextual variables to work volition, career adaptability, and decent work (Duffy et al., 2016a). This framework has received increasing attention in recent years, perhaps due in part to its empirical assumptions, which is also why we selected it as the theoretical framework in this study.

The current PWT model primarily stems from a North American perspective and a Western cultural framework that highlights individualism; therefore, the applicability of the theory may be limited in the extent to which it is generalizable outside of North America and outside of an individualistic cultural framework in particular. Thus, researchers and practitioners must exercise caution when extending these findings to other nations and regions of the world (Duffy et al., 2016a), which means that more research with non-US and non-Western samples is needed to extend the knowledge of work volition and understand the different cultural, social, and familial influences (Kim et al., 2018). Therefore, conducting research in China, an Eastern country with a typical collectivistic culture, would be meaningful, as the findings could significantly broaden the understanding of work volition and address the abovementioned critical research gap related to this theory.

## 3. Literature review and research hypotheses

### 3.1. Economic constraints and work volition

Economic constraints are primarily defined by limited economic resources (e.g., household income and family wealth), which represent a critical barrier to securing decent work, and are considered to be a significant negative predictor of work volition (Duffy et al., 2016a). This finding has been supported by an expansive body of research conducted with different participants from different countries. For example, Cheung et al. (2020) indicated that constraints will be negatively related to work volition among university students in the United States and Hong Kong. Kim and Allan (2021) also found that economic constraints significantly influence the work volition of employees in the United States. Longitudinal studies also demonstrated that students with greater economic deprivation were less likely to feel volitional in their career decision-making (Allan et al., 2019). Some researchers attributed this to differences in resources. Specifically, parents who have fewer economic resources and are less educated often have difficulties imparting essential or necessary knowledge regarding educational and career pathways to their children that could help in the career-planning process (Blustein et al., 2002). Based on these previous studies, we established our first hypothesis.

**Hypothesis 1:** Economic constraints are negatively associated with work volition.

### 3.2. Minzu (ethnic identity) and work volition

Researchers in vocational psychology have taken a leading role in studying how race/ethnicity may ultimately affect the ability to freely choose the careers one wants to pursue (Duffy et al., 2012). Although a few studies found no significant differences in work volition between the majority and minority racial groups (Duffy et al., 2014), a robust body of literature supports the finding that work volition is significantly higher in the majority racial groups compared with the minority racial groups (e.g., Luzzo and McWhirter, 2001; Jadidian and Duffy, 2012). This is largely due to the differences in status between the majority and minority racial groups and consequent marginalized experiences. Marginalization represents the relegation of people (or groups of people) to a less powerful or included position within a society, and it is also considered to be a significant negative predictor of work volition. Racial or ethnic minority groups experience a form of marginalization; accordingly, racial or ethnic minorities tend to have less work volition (Duffy et al., 2016a).

Despite the fact that there are one majority and fifty five minority ethnic groups in China, the meaning of the “ethnic group” in the Chinese context differs from that in other countries. China is a unitary 56-nation country. Thus, members of the Han majority ethnic group and all ethnic minorities have interacted with each other throughout the history of China to integrate into one nation. Moreover, all ethnic minorities are officially recognized by the Chinese government, and their equal rights are protected by the national constitution; therefore, no groups experience marginalization as described above. To avoid misperceptions that can arise with words such as “ethnicity” and “minority,” which can imply assumptions of marginalization, we use the Chinese word *minzu*, which includes people in all minority groups and the Han majority (Yuan et al., 2020). Taking the above arguments together, we established the second hypothesis.

**Hypothesis 2:** Minzu is not related to work volition.

### 3.3. Hukou and work volition

The hukou system was implemented initially in Chinese cities in 1951 and extended to rural areas in 1955 (Lu and Wan, 2014), which categorized citizens into urban and rural residents of a particular location (Afridi et al., 2015) as follows: individuals have a rural hukou if the permanent residence identity is registered in rural areas, while they have an urban hukou if it is registered in cities (Tani, 2016). The system was designed to serve multiple state interests as a part of a larger economic and political system (e.g., block rural-urban migration, Chan and Zhang, 1999). The hukou system is connected to people's access to state-provided benefits and opportunities; therefore, it significantly affects the daily lives of people in many aspects (Chan and Zhang, 1999). On average, those with a rural hukou are socioeconomically worse off than those with an urban hukou, and the gap between hukou statuses is also reflected in the allocation of resources and services such as education, health care, and pensions (Knight et al., 2009; Afridi et al., 2015).

Regarding work volition as conceptualized in the present study, Wang et al. (2019) stated that access to opportunities in China is significantly influenced by external factors such as hukou status. Guo et al. (2013) also noted that the hukou category, a unique indicator of socioeconomic status, largely determines the eligibility and access of a person to various social and economic benefits, and an urban hukou status is associated with privileges. Kukla et al. (2016) further indicated that supported employment personnel from rural programs perceived significantly more barriers to working successfully compared with urban personnel, particularly in terms of access to services and a range of job-related factors. Yu et al. (2014) found that college students from urban areas are more familiar with the basic living patterns of cities, so their anxiety is low; however, college students from rural areas with strong urban–rural differences will have strong anxiety when they plan to seek employment in cities, resulting in decreased confidence (Klassen and Marx, 2020; Zheng et al., 2022). Based on these previous studies, we can infer that children from rural areas face worse economic conditions and receive fewer learning resources compared with children from urban areas, which will result in rural students being unable to obtain vocational training and vocational information comparable to that of urban students. Simply put, we established the third hypothesis.

**Hypothesis 3:** Hukou is related to work volition.

Several studies documented a negative link between economic constraints and work volition; however, hukou may provide a new and more detailed perspective on this relationship. Cheng (2018) criticized Bourdieu's theory of cultural reproduction and, based on qualitative interviews, proposed the concept of cultural capitals of the underclass. In short, he stated that, although children living in rural areas do not have inherent objective advantages, their own bottom-level situation naturally produces positive qualities such as a strong work ethic and perseverance. In addition, the support parents give their children under difficult economic conditions will also inspire those children to strive for progress. Thus, we posit that undergraduates from rural areas may have more valuable responses to their own economic constraints and stronger determination to seek to overcome difficulties and change their destiny. However, people living in urban areas have higher incomes and living standards; thus, they may have a lower tolerance for economic constraints, which may lead to more negative outcomes when they encounter them. Based on this study, we established the fourth hypothesis.

**Hypothesis 4:** Hukou can moderate the relationship between economic constraints and work volition. To be specific, the negative relationship between economic constraints and work volition is weaker for people from rural areas, while it is stronger for people from urban areas.

### 3.4. Zhongyong thinking and work volition

Zhongyong thinking, one of the most influential Chinese thinking styles, refers to the practitioner carefully considering things from different perspectives and choosing a way that can integrate the individual and the situation (Wu and Lin, 2005). Previous studies found that people with high levels of Zhongyong thinking have a greater capacity for multiple-signal processing than those with low levels, indicating that they have a well-developed sense of the interests of conflicting parties and process information in an integrated manner (Chang and Yang, 2014). Therefore, it is easier for them to make wise decisions (Wei and Wang, 2020). According to the above definition and literature, we posit that the higher the level of Zhongyong thinking of individuals, the more they can consider the factors involved in making career decisionsand the higher their perceived ability to make occupational choices. Therefore, we established the fifth hypothesis.

**Hypothesis 5:** Zhongyong thinking is related to work volition.

Zhongyong thinking can help describe a more delicate relationship between economic constraints and work volition, although it has been rarely examined in previous literature. The primary aim of Zhongyong thinking is to find an optimal point of balance and avoid excess in whatever direction one takes. It proposes that all things or phenomena have corresponding limits that define their proper, optimal states or conditions (Cheung et al., 2003); thus, this should apply to the relationship between economic constraints and work volition. Zhongyong thinking emphasizes taking a holistic perspective on a situation (Yao et al., 2010) and avoiding excess; thus, the higher the level of Zhongyong thinking of individuals, the more they can perceive their own economic constraints in the real world and avoid setting unreasonable expectations, which could result in lower work volition. In other words, the lower their level of Zhongyong thinking, the more difficult it is for individuals to combine various factors; thus, the relationship between economic hardship and career decision-making may be more stable. Therefore, we established the sixth hypothesis.

**Hypothesis 6:** Zhongyong thinking can moderate the relationship between economic constraints and work volition. The negative relationship between work volition and economic constraints is stronger for people with high levels of Zhongyong and weaker for those with low levels of Zhongyong thinking.

**Figure 1:**
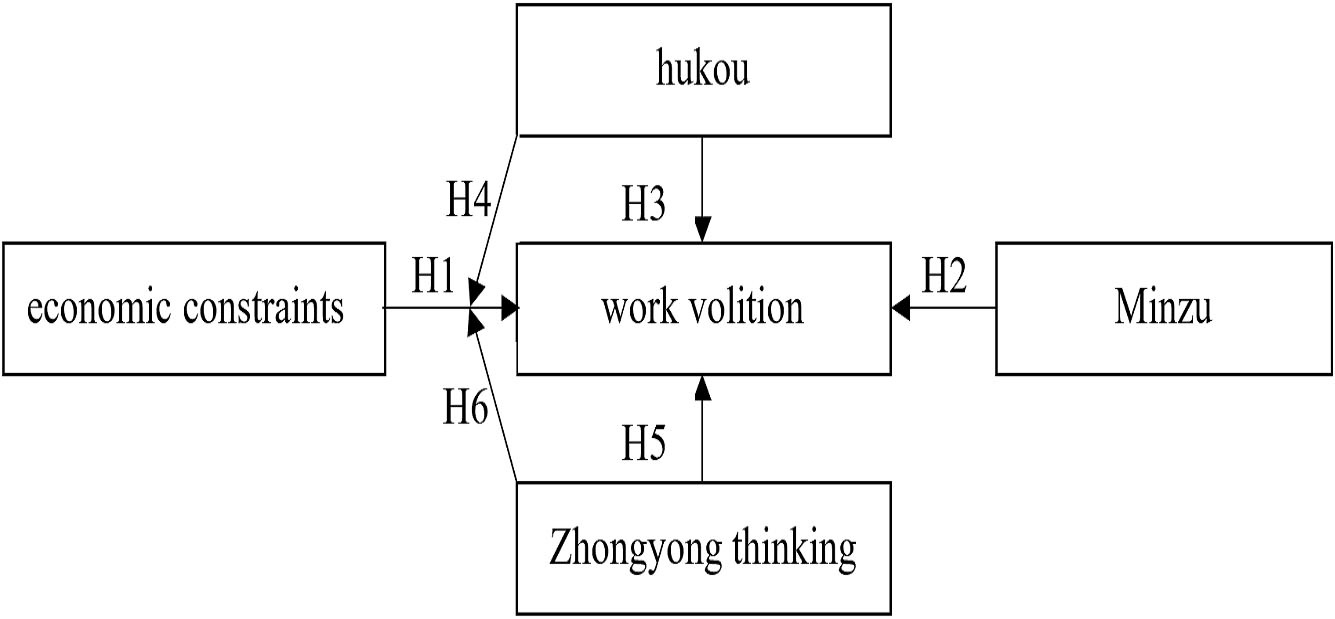
The hypothesis model.

The hypothesized model of this study is shown in Figure 1.

### 3.5. The present study

Based on prior research on work volition, the goal of the current study is to explore how economic constraints, minzu, hukou, and Zhongyong thinking, are related to work volition, and how hukou and Zhongyong thinking moderate the relationship between economic constraints and work volition. Moreover, we chose college students as participants for two reasons. On the one hand, college students are likely at critical points in their decision-making process (Duffy et al., 2016b), and making a career decision is an important career issue for them (Cheung et al., 2020). On the other hand, college students are likely to face increasing barriers to achieving their ultimate career goals (Duffy et al., 2012; Cheung et al., 2020), such as mobility constraints (Wei et al., 2022), although they are considered at the top of the career privilege pyramid and possess more freedom and volition than others when making their career choice because of professional competence, qualifications, and generic skill sets (e.g., analytical skills and critical thinking) based on on-campus training and exposure to extracurricular programs (Cheung et al., 2020). Therefore, it is necessary to examine the state of the population; however, previous studies have been largely restricted to working adults (Wei et al., 2022).

## 4. Method

### 4.1. Participants

A total of 3,500 undergraduates from ten provinces (i.e., Guangxi, Guizhou, Jilin, Qinghai, Shandong, Chongqing, Jiangsu, Anhui, Sichuan, and Xinjiang) in mainland China were recruited and were asked to complete questionnaires. Due to poor response quality, 505 (14.43%) questionnaires were excluded from further analysis. The final sample included 2,995 participants aged from 17 to 26 years (M = 19.86, SD = 1.72), with 1,298 (43.30%) men and 1,697 (56.70%) women; 2,305 (77.00%) Han and 690 (23.00%) ethnic minority; 2,305 (77.00%) from rural areas and 690 (23.00%) from urban areas; and 1,140 (38.10%) freshmen, 675 (22.50%) sophomores, 659 (22.00%) juniors, and 521 (17.40%) seniors. A total of 4,861 participants reported monthly household incomes of < 2,000 RMB (6.20%), 938 of 2,000–4,000 RMB (31.30%), 604 of 4,000–6,000 RMB (20.20%), 380 of 6,000–8,000 RMB (12.70%), 234 of 8,000–10,000 RMB (7.80%), and 353 of more than 10,000 RMB (11.80%).

### 4.2. Instruments

#### 4.2.1. Economic constraints

Economic constraints were assessed using the 5-item Economic Constraints Scale (ECS) for students, which was developed by Duffy et al. (2019). Sample items include “Throughout most of my life, I have struggled financially” and “For most of my life, I have not felt financially stable”. Items were rated on a 7-point scale ranging from 1 (strongly disagree) to 7 (strongly agree), with higher average scores of the five items indicating higher levels of economic constraints. The estimated internal consistency reliability of scale scores in the present study was 0.96.

#### 4.2.2. Work volition

Work volition was assessed using the Work Volition Scale—Student Version (WVS-SV; Duffy et al., 2012). The WVS-SV is composed of 16 items, including two dimensions, namely, volition (e.g., Discrimination will not affect my ability to choose a job) and constraints (e.g., What I want has little impact on my future job choices). Items were rated on a 7-point scale ranging from 1 (extremely disagree) to 7 (extremely agree), with higher average scores of total items indicating higher levels of work volition. The estimated internal consistency reliability of scale scores in the present study was 0.77.

#### 4.2.3. Zhongyong thinking

Zhongyong thinking was measured using a Zhongyong Thinking Style Scale, which was developed by Wu and Lin (2005). It is composed of 13 items that are divided into three dimensions, namely, multi-thinking (e.g., When discussing opinions, I will take into account the multiple opinions in disputes), holism (e.g., I will adjust my original thinking after considering the opinions of others), and harmoniousness (e.g., I usually express conflicting opinions euphemistically). Items were rated on a 7-point scale ranging from 1 (extremely disagree) to 7 (extremely agree), with higher average scores of total items indicating higher levels of Zhongyong thinking. The estimated internal consistency reliability of scale scores in the present study was 0.97.

### 4.3. Procedure

Data were collected through Wenjuanxing, a popular online survey platform in China, between 1 February and 12 October 2022. The information sheet and the link to the survey were sent to potential participants *via* WeChat, a widely used instant messaging and social media application in China. Participants indicated their consent by completing the questionnaire. Participation was anonymous. It took up to 10 min to complete the survey.

### 4.4. Data analysis

Using SPSS 21.0, we first conducted two hierarchical regression analyses to investigate the relationship among economic constraints, minzu, hukou, and Zhongyong thinking, and the way that hukou and Zhongyong thinking moderate the relationship between economic constraints and work volition. We tested hierarchical regression in two steps. In step 1, we added all predictors, i.e., economic constraints, minzu, hukou, and Zhongyong thinking. In step 2, we added the interaction terms (economic constraints and hukou, economic constraints, and Zhongyong thinking), which we created with centered variables to reduce multicollinearity. Then, we performed a formal F-test as mentioned earlier to further confirm their relationship.

## 5. Results

### 5.1. Preliminary analyses

A number of preliminary analyses were conducted before formal data analysis. First, to check the quality of the data, skewness and kurtosis were calculated for all continuous variables. Kurtosis and skewness are between −0.53 and 0.37 and between −0.40 and 0.18, respectively, denoting that the shape of the data distribution in the study may not be severely nonnormal because of the absolute values of kurtosis ≤ 10.0 and skewness ≤ 3.0 (Kline, 2015). Second, we explored the means, standard deviations, and correlations with all variables. As seen in Table 1, the economic constraints was significantly positively correlated with minzu and Zhongyong thinking and significantly negatively correlated with hukou and work volition; minzu was significantly negatively correlated with hukou and work volition; hukou was significantly positively correlated with Zhongyong thinking and work volition; and Zhongyong thinking was significantly positively correlated with work volition.

**Table 1:** Descriptive statistics and correlations.

**Variables**	**1**	**2**	**3**	**4**	**5**
1. Economic constraints	1				
2. Minzu	0.05^*^	1			
3. Hukou	−0.31^***^	−0.14^***^	1		
4. Zhongyong thinking	0.14^***^	0.01	0.06^**^	1	
5. Work volition	−0.35^***^	−0.05^**^	0.17^***^	0.20^***^	1
*M*	4.13	1.23	1.23	5.13	4.28
*SD*	1.46	0.42	0.42	0.95	0.65

^*^p < 0.05,

^**^p < 0.01,

^***^p < 0.001. Rural hukou = 1, Urban hukou = 2; Han = 1, Ethnic minority = 2.

### 5.2. Hierarchical regression and *F*-test

First, we examined the predicting effect of economic constraints, minzu, hukou, and Zhongyong thinking on work volition in Step 1. Table 2 shows the result of this hierarchical regression: Economic constraints significantly negatively predict work volition, while Zhongyong thinking positively predicts work volition, but minzu and hukou could not significantly predict work volition. Therefore, Hypotheses 1, 2, and 5 were supported, but Hypotheses 3 were rejected. Second, we examined the moderating effect of hukou and Zhongyong thinking on the relationship between economic constraints and work volition in Step 2. We added interaction terms and found that economic constraints by hukou and economic constraints by Zhongyong thinking significantly predicted work volition. This supports Hypotheses 4 and 6, which posit that hukou and Zhongyong thinking play important roles in determining the level of work volition of individuals when facing economic constraints. Notably, the negative effect (economic constraints – work volition) is quite large statistically compared with the coefficient for the interaction term (economic constraints ^*^ hukou – work volition). The negative (economic constraints – work volition) and positive (Zhongyong thinking – work volition) effects are quite large statistically compared with the coefficient for the interaction term (economic constraints ^*^ Zhongyong thinking – work volition).

**Table 2:** Hierarchical regression examining the relationship among economic constraints, minzu, hukou, Zhongyong thinking, and work volition.

**Variable**	**β**	***B***	***SE B***	***p***	***R***	***R^2^***	***R^2^Δ***	***F***
**Step 1**								
Overall model					0.43	0.19	0.18	170.02
Economic constraints	−0.37	−0.16	0.01	0.000				
Minzu	−0.03	−0.04	0.03	0.086				
Hukou	0.03	0.03	0.01	0.051				
Zhongyong thinking	0.25	0.17	0.01	0.000				
**Step 2**								
Overall model					0.45	0.20	0.20	126.10
Economic constraints	−0.19	−0.08	0.02	0.000				
Minzu	−0.03	−0.05	0.03	0.051				
Hukou	0.01	0.01	0.01	0.497				
Zhongyong thinking	0.24	0.17	0.01	0.000				
Economic constraints ^*^ Hukou	−0.10	−0.04	0.01	0.000				
Economic constraints ^*^ Zhongyong thinking	−0.18	−0.04	0.01	0.000				

**Figure 2:**
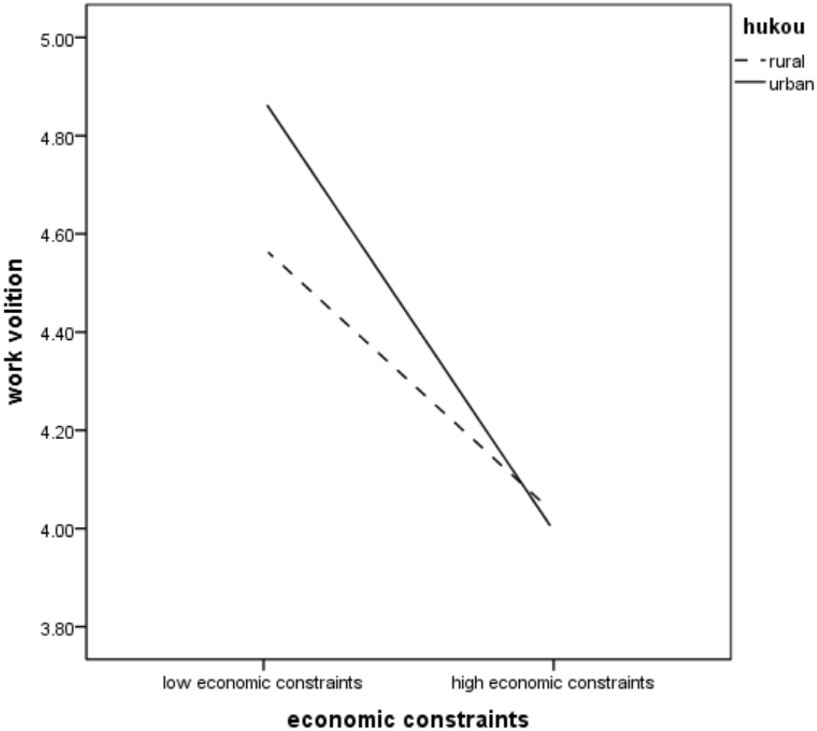
Hukou moderating the relation between economic constraints and work volition.

To further confirm the relationship between these variables, we performed an *F*-test that included hukou, Zhongyong thinking, and economic constraints. A two-factor *F*-test was conducted with economic constraints and hukou as independent variables and work volition as dependent variables. The results showed that the main effect of economic constraints was significant (*F* = 148.62, *p* < 0.001), and the work volition of people with low economic constraints was significantly higher than that of people with high economic constraints. The main effect of hukou was significant (*F* = 5.20, *p* < 0.05), and the work volition of students from rural areas was significantly higher than that of students from urban areas. The interaction effect of economic constraints and hukou was significant (*F* = 8.46, *p* < 0.01), which further supports Hypothesis 4. As Figure 2 shows, the relationship between economic constraints and work volition was more weakly negative for people from rural areas, whereas it was more strongly negative for people from urban areas.

**Figure 3:**
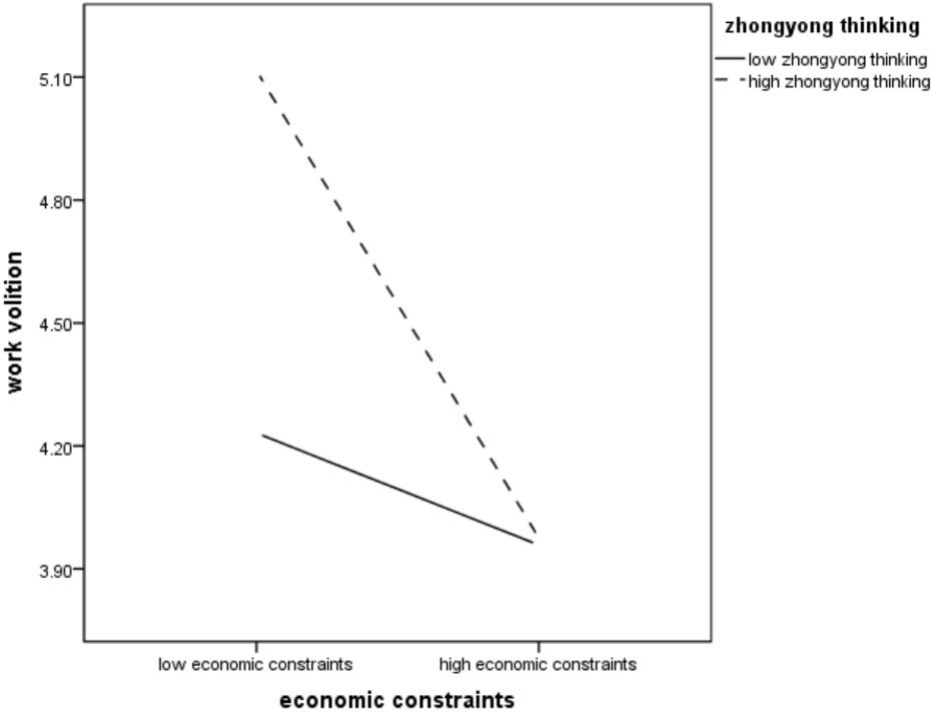
Zhongyong thinking moderating the relation between economic constraints and work volition.

At the same time, another two-factor *F*-test was conducted with economic constraints and Zhongyong thinking as independent variables and work volition as dependent variables. The results showed that the main effect of economic constraints was significant (*F* = 73.19, *p* < 0.001), and the work volition of people with low economic constraints was significantly higher than that of people with high economic constraints. The main effect of Zhongyong thinking was significant (*F* = 29.78, *p* < 0.001), and the work volition of students with a high level of Zhongyong thinking was significantly higher than that of students with a low level of Zhongyong thinking. The interaction effect of economic constraints and Zhongyong thinking was significant (*F* = 27.57, *p* < 0.001), which further validates Hypothesis 6. Figure 3 indicates that the negative relationship between work volition and economic constraints is stronger for people with high levels of Zhongyong thinking and weaker for those with low levels of Zhongyong thinking.

## 6. Discussion

The results of this study showed that the average work volition of Chinese undergraduates is 4.26, lower than the work volition of people from other countries (e.g., Duffy et al., 2012), which can be explained as follows. Economic constraints and Zhongyong thinking were found to significantly negatively and positively predict work volition, respectively, while minzu and hukou could not significantly predict work volition. Moreover, hukou and Zhongyong thinking were found to have moderating effects on the relationship between economic constraints and work volition. For people from rural areas, the relationship between economic constraints and work volition was more weakly negative, whereas, for people from urban areas, it was strongly negative. In addition, the relationship between economic constraints and work volition was more weakly negative for those with low levels of Zhongyong thinking, whereas it was more strongly negative for those with high levels of Zhongyong thinking.

In line with past studies (e.g., Duffy et al., 2019; England et al., 2020; Kim and Allan, 2021; Kim et al., 2022), our results support the basic assumption that economic constraints have a negative association with work volition, showing that participants who experienced more economic barriers were less likely to report high levels of work volition. Furthermore, the negative effect (economic constraints – work volition) was quite statistically large compared with the coefficient for the interaction term (economic constraints × hukou – work volition; economic constraints – Zhongyong thinking – work volition), indicating the strength of the effect of economic constraints. A possible explanation for this finding is that economic conditions may directly influence resources that individuals can obtain, and the latter, in turn, influences work volition. Researchers found differences in external educational resources and barriers according to the socioeconomic status of people. Adults with a higher socioeconomic status appear to have greater access to vocationally and personally salient resources. Although these individuals can face some barriers, the frequency and severity of their educational external barriers are less overt and influential, whereas their counterparts with lower socioeconomic status have indicated that, while their schools have guidance offices and other resources, these services are less available and helpful, and they experience more pervasive and less easily remedied educational barriers. Moreover, researchers also found that relational resources also vary according to socioeconomic status, especially in terms of parental resources. Adults with higher socioeconomic status are more likely to have parents who are not only supportive and explorative but also instrumental in their career planning; thus, they are more likely to receive instrumental and agentic help regarding career decision-making. In contrast, adults with a lower socioeconomic status do not receive substantive instrumental help from their parents, who have less economic resources and education, nor do they receive encouragement, support, or offer to provide career-planning or decision-making advice (Blustein et al., 2002). Overall, individuals with a higher socioeconomic status enjoy more educational and relational resources; thus, they tend to have a clearer sense of their career goals, are aware of what they need to do to achieve them, and have fewer constraints or a more positive perception to overcome possible constraints compared with individuals with lower socioeconomic status (Blustein et al., 2002).

Supporting our hypothesis, minzu was not found to be related to work volition, which differs from the findings of most studies on ethnic identity, a different expression, in the field (e.g., Luzzo and McWhirter, 2001). However, as previously noted, this difference may be at least partly explained by the status of minzu in China. Duffy et al. (2018) reported that the career development and workplace functioning of various racial or ethnic minority groups may be influenced by their exposure to racial or ethnic marginalization, and this will negatively predict work volition, based on PWT (Duffy et al., 2016a). For example, ethnic minority students were found to be more likely to perceive career-related barriers directly associated with their ethnicity, including the expectation that they will experience negative comments about and discrimination based on their ethnic or racial background (Luzzo and McWhirter, 2001). In contrast, each minzu has a different culture and conventions in China, but all enjoy the same rights. The Chinese government has even promulgated many measures to guarantee and promote the employment of people from different ethnic groups (excluding the Han majority group); thus, no groups experience marginalization. Thus, it is understandable why minzu is not significantly related to work volition in China.

Contrary to our hypotheses suggesting a link between hukou and career obstacles (Wang et al., 2019), these two variables were not significantly related, which is in line with the findings of Li et al. (2008) and Hu (2012). We attribute these results to the shrinking gap and changeable characteristics between urban and rural areas. The Chinese government has implemented several initiatives to address the inequality associated with hukou since 1978 (Tani, 2016), thereby sharply reducing rural poverty (Knight et al., 2009). Moreover, those who remain in rural areas have more land and other resources and can expand their scale of production when more rural laborers move into cities in the process of urbanization, which also reduces inequity between hukou categories (Lu and Wan, 2014). Regarding changeable characteristics, unlike social class or social status which are relatively stable and difficult to change (Cheung et al., 2020), the gap between hukou categories has become smaller (Chan and Zhang, 1999). Furthermore, mobility derived from a market-oriented economy and urbanization has greatly challenged the basis of the traditional hukou registration system and forced the government to adjust its policies, including allowing migrants to change their hukou category after they have been employed in a city for a certain period (Chan and Zhang, 1999; Lu and Wan, 2014). Thus, mobility constraints (Wei et al., 2022) have been taken seriously and largely resolved. Therefore, the finding that hukou does not have a negative impact of classism on work volition is understandable (e.g., Wang et al., 2019; Kim and Allan, 2021), based on the analysis above.

Perhaps the most intriguing finding in the present study is that hukou did not show a significant direct effect on work volition but could moderate the relationship between economic constraints and work volition. A possible explanation for this is related to the cultural capital of the underclass for rural students. Cheng (2018) posited that the lack of resources and economic affluence faced by rural students are an important foundation for the cultural capital of this group. Specifically, their harsh living environment forces them to study hard and become steadfast, hardworking, tenacious, and persistent. Parental support and poor living conditions will increase the motivation of rural students to continue to make progress on a moral level. In addition, the limitations of the living environment and the abilities of parents make rural students have more faith than urban students in the beliefs conveyed by their school, e.g., that reading can change the fate of a person. Thus, it can be inferred that the impact of economic constraints on rural students is more likely to be motivational than restrictive. When making occupational choices, rural students tend to face difficulties with positive qualities, such as optimism, gratitude, confidence, and persistence, shaped by financial hardship, supportive parental behaviors, and a strong belief in the philosophy of their school, which effectively reduces the negative impact of economic constraints on work volition. This is also partially consistent with the findings of Chaves et al. (2004) and Cheung et al. (2020) who reported that undergraduate students would perceive a high capacity in career decision-making despite the presence of constraints when they possess personal resources, which is related to familial aspirations for children in rural areas.

Verifying our hypothesis, Zhongyong thinking is statistically significant in relation to work volition, which can be interpreted based on the essence of Zhongyong thinking. Zhongyong thinking emphasizes the way of thinking in which individuals integrate external conditions and internal needs in a specific situation and take appropriate actions (Wu and Lin, 2005). Thus, those with higher levels of Zhongyong thinking are more likely to acknowledge their own strengths and weaknesses and suitability for potential jobs and are therefore more confident in their career choices.

As we hypothesized, Zhongyong thinking was found to have a moderating effect on the relationship between economic constraints and work volition. People with low levels of Zhongyong thinking had a weaker negative relationship between economic constraints and work volition, whereas people with high levels of Zhongyong thinking had a stronger negative relationship between economic constraints and work volition. This can be attributed to two factors. First, unlike instrumental-rational action, Zhongyong thinking is not intended to simply maximize efficiency or generate as much utility as possible but instead represents a “tempered mode” (Cheung et al., 2003). People with Zhongyong thinking must fully assess the economic constraints they face, place them in an optimal situation, and make rational work volition based on the level of economic constraints, which is especially true among people with high levels of Zhongyong thinking. Those with high levels of Zhongyong thinking strive to also have relatively high work volition when facing fewer economic constraints or having sufficient capacity and resources; however, they may reduce their perception of work volition to achieve optimization if the economic constraints they face are too serious or decisive. This fully embodies the characteristics of the pursuit of achieving a state of balance and harmony in Zhongyong thinking. Conversely, people with low levels of Zhongyong thinking are less likely to have the flexibility to change their work volition based on the economic constraints they face. Second, from the perspective of emotion, negative economic situations can lead individuals to experience negative emotions such as anxiety and stress, and the latter will negatively affect the career decision-making self-efficacy of an individual (Kuang et al., 2011). People with high levels of Zhongyong thinking are more concerned about their individual situation (Li et al., 2021). Therefore, they are also more sensitive to the adverse effects of their economic situation, which was reflected in the present study by individuals with greater economic constraints reporting significantly lower work volition than those with fewer economic constraints. However, because they pay less attention to their individual environment, those with low levels of Zhongyong thinking can have a more stable attitude toward economic difficulties; thus, they showed a non-significant change in work volition in the process of moving from low to high economic constraints. We believe that different levels of Zhongyong thinking have different working mechanisms in the relationship between economic constraints and work volition; thus, in the present study, high levels of Zhongyong thinking had an “enhancing” effect, while low levels of Zhongyong thinking had a “buffering” effect.

### 6.1. Practical implications

To the best of our knowledge, our study is the first to examine the effects of certain variables on work volition based on a common and specific culture (i.e., Chinese situation and culture). Moreover, we not only confirmed the relationship between economic constraints and work volition in previous studies but also found relationships between new variables such as minzu, hukou, and Zhongyong thinking and work volition, which may have important implications for counselors working with college students.

At the commonality level, consultants should be aware of variables that affect work volition that are prevalent across cultures in vocational psychology. For example, economic constraints are important because they can inform counselors of what relevant actions to take quickly and efficiently, including exploring how economic constraints may reduce the sense of choice of an individual in work volition, thereby increasing financial subsidies and improving the distribution of resources to groups who face economic constraints.

At the specificity level, although PWT is believed to be excellent in capturing the predictors of work volition, it does not sufficiently meet the needs of acknowledging the work volition of people living in a special context, especially in non-Western cultures. Thus, counselors should be encouraged to explore specific factors that affect work volition among specific populations, such as minzu, hukou, and Zhongyong thinking, although there are certain difficulties. Moreover, consultants should also pay attention to the cross-cultural significance of some variables. For example, although ethnic minorities tend to have lower work volition in many countries (e.g., Luzzo and McWhirter, 2001), this was not found to be the case in China. This means that counselors need to critically examine the role of certain variables according to the specific situation to effectively implement interventions to bolster work volition.

### 6.2. Limitations and future directions

The results of the current study need to be considered in light of a number of limitations, all of which inform directions for future research. First, it used a cross-sectional design in which participants were recruited during a single time point, which means that it precludes the possibility of determining causality between the study variables. Future research should determine the causal mechanisms of these variables using a longitudinal design or experimental methods. Second, Chinese undergraduates were recruited as participants, and although the selection of this group is meaningful and representative, it is important to acknowledge that the population will limit the extent to which the results can be generalized. Future research should boost the generalizability of the results by replicating the study with a more diverse sample. Third, the moderating effects of hukou and Zhongyong thinking on the relationship between economic constraints and work volition were low and only explained 1% of the variance of work volition; therefore, caution is needed when interpreting the results. Future studies should verify the accuracy of the moderating effects by increasing the sample size and strictly controlling the study conditions.

## 7. Conclusion

Research on work volition has burgeoned in recent years. Although the study has its limitations, it aimed to expand to this growing literature stream by supporting old propositions (e.g., economic constraints were related to work volition) while exploring new ones (Zhongyong thinking and hukou play more important roles in determining the reported level of work volition facing economic constraints) with Chinese undergraduates. The findings also provide practical implications for counselors to improve work volition in a particular population by focusing on both common variables (validated in both Eastern and Western cultures) and specific variables (validated in a specific culture).

## Data availability statement

The raw data supporting the conclusions of this article will be made available by the authors, without undue reservation.

## Ethics statement

The studies involving human participants were reviewed and approved by the School of Education, Minzu University of China. The Ethics Committee waived the requirement of written informed consent for participation.

## Author contributions

Conceptualization, formal analysis, data curation, and writing—review and editing: LH and XB. Methodology: XB. Software, resources, writing—original draft preparation, and project administration: LH. Validation and visualization: WL. Investigation: LH and WL. Supervision: XB and WL. All authors have read and agreed to the published version of the manuscript.
